# Unveiling the Role of Ge in CZTSSe Solar Cells by Advanced Micro‐To‐Atom Scale Characterizations

**DOI:** 10.1002/advs.202305938

**Published:** 2024-02-11

**Authors:** Jialin Cong, Mingrui He, Jun Sung Jang, Jialiang Huang, Karen Privat, Yi‐Sheng Chen, Jianjun Li, Limei Yang, Martin A. Green, Jin Hyeok Kim, Julie M. Cairney, Xiaojing Hao

**Affiliations:** ^1^ Australian Centre for Advanced Photovoltaics School of Photovoltaic and Renewable Energy Engineering University of New South Wales Sydney New South Wales 2052 Australia; ^2^ Optoelectronic Convergence Research Center Department of Materials Science and Engineering Chonnam National University Gwangju 61186 South Korea; ^3^ Electron Microscope Unit Mark Wainwright Analytical Centre University of New South Wales Sydney New South Wales 2052 Australia; ^4^ Australian Centre for Microscopy and Microanalysis (ACMM) The University of Sydney Sydney New South Wales 2006 Australia; ^5^ School of Civil and Environmental Engineering University of Technology Sydney Sydney New South Wales 2007 Australia

**Keywords:** Ge incorporation, kesterite, micro‐to‐atomic‐scale characterization, underlying mechanism

## Abstract

Kesterite is an earth‐abundant energy material with high predicted power conversion efficiency, making it a sustainable and promising option for photovoltaics. However, a large open circuit voltage V_oc_ deficit due to non‐radiative recombination at intrinsic defects remains a major hurdle, limiting device performance. Incorporating Ge into the kesterite structure emerges as an effective approach for enhancing performance by manipulating defects and morphology. Herein, how different amounts of Ge affect the kesterite growth pathways through the combination of advanced microscopy characterization techniques are systematically investigated. The results demonstrate the significance of incorporating Ge during the selenization process of the CZTSSe thin film. At high temperature, the Ge incorporation effectively delays the selenization process due to the formation of a ZnSe layer on top of the metal alloys through decomposition of the Cu‐Zn alloy and formation of Cu‐Sn alloy, subsequently forming of Cu‐Sn‐Se phase. Such an effect is compounded by more Ge incorporation that further postpones kesterite formation. Furthermore, introducing Ge mitigates detrimental “horizontal” grain boundaries by increasing the grain size on upper layer. The Ge incorporation strategy discussed in this study holds great promise for improving device performance and grain quality in CZTSSe and other polycrystalline chalcogenide solar cells.

## Graphical abstract

With a thin Ge nanolayer deposited on a prealloyed metallic stacked precursor, the formation of ZnSe is more favorable over Cu_2_Se due to larger negative Gibbs free energy for ZnSe when selenium reacts with Cu‐Zn alloy based on thermodynamic calculation. Cu from decomposed Cu‐Zn alloy exhibited a tendency to alloy with metal Sn, forming Cu‐Sn alloy and effecting subsequent kesterite formation pathway.

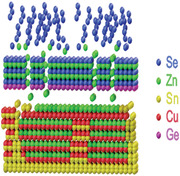

## 1 Introduction

Kesterite Cu_2_ZnSn(S,Se)_4_ (CZTSSe) is a promising candidate for thin film solar cells due to its high absorption coefficient of over 10^4^ cm^−1^ and tunable bandgap.^[^ ^1^, ^2^, ^3^ ^]^ It comprises earth‐abundant and low toxicity elements, making it an environmentally friendly alternative to traditional thin film solar cell materials such as Cu(In,Ga)Se_2_ (CIGS). Despite its potential, the power conversion efficiency (PCE) of the champion CZTSSe has yet to reach high efficiency even at the laboratory level, with the current champion cell achieving a PCE of 14.9%.^[^ ^4^ ^]^ The main obstacle for CZTSSe solar cells, which hinders further performance enhancement, is a significant open circuit voltage (V_oc_) deficit. The maximum theoretical V_oc_ determined using the Shockley‐Queisser approach (V_oc_^SQ^) at the corresponding bandgap E_g_ is calculated using the relation V_oc_^SQ^ (V) = 0.932∗E_g_ (eV) − 0.167, where E_g_ is derived from the dEQE/dλ curve,^[^ ^5^ ^]^ where EQE is external quantum efficiency and λ is wavelength. To date, the highest V_oc_/V_oc_^SQ^ value of kesterite solar cell is just above 60%, which lags far behind the corresponding value for CIGS (over 85%).^[^ ^6^ ^]^ One origin of the large V_oc_ deficit is attributed to abundance of intrinsic defects in the bulk and at interfaces, especially Sn‐related defects. As a multivalent element (+2, +4 valence states), Sn is known to introduce deep level defects, including Sn_Zn_ and [2Cu_Zn_ +Sn_Zn_] defect clusters, which significantly enhance non‐radiative charge carrier recombination, and therefore negatively impact V_oc_.^[^ ^7^, ^8^, ^9^ ^]^ Furthermore, an unfavorable morphology, which includes the formation of small grains and pinholes at the bottom of the absorber, results in poor carrier transport at the back contact and increased charge recombination, leading to another V_oc_ loss mechanism. Recently, our research group has found that the recombination velocity at grain boundaries in kesterite solar cells is one of the main constraints on device performance.^[^ ^10^ ^]^ Therefore, it is ideal to control the crystallization process to form a large‐grained, columnar‐like structure which would improve the morphology of kesterite by directly reducing the number of detrimental grain boundaries. Currently, more emphasis is consequently being placed on enhancing the crystalline quality and reducing defects in terms of improving efficiency of kesterite solar cell.

Substituting Sn with Ge has proven to be an effective approach to inhibit deep defect Sn_Zn_ and associated carrier recombination.^[^ ^9^, ^11^, ^12^ ^]^ CZTSSe solar cells exhibit improved electronic properties by either alloying or doping Ge.^[^ ^13^, ^14^, ^15^ ^]^ A Ge‐alloyed Cu_2_Zn(Sn_1−x_Ge_x_)Se_4_ thin film solar cell with 12.3% PCE was reported^[^ ^16^ ^]^ showing reduced band tailing and an improved V_oc_ and fill factor (FF) due to reduced carrier recombination at the absorber/buffer interface and/or in the space charge region (SCR). Giraldo et al.^[^ ^14^ ^]^ demonstrated a kesterite solar cell with up to 11.8% efficiency by introducing an ultrathin Ge nanolayer at the rear region. The presence of Ge prevents drastic compositional fluctuations during the annealing process and alters the reaction pathway of the kesterite. A CZTSSe solar cell with a GeO_2_ layer on the Mo substrate introduced by the spin‐coating method achieved up to 13.14% PCE and a greatly improved V_oc_ due to reduced bulk defects, band tailing, and an optimized back interface.^[^ ^15^ ^]^ Our previous research on double cation (Ge, Cd) incorporation suggested that increasing the presence of Ge could enhance p‐type doping and improve bulk conductivity. This, in turn, led to superior V_oc_, short‐circuit current density (J_sc_), and FF, significantly improving the overall power conversion efficiency.^[^ ^17^ ^]^ In addition to improved carrier transportation and reduced deep‐level defects, enlarged grain size, and better crystalline quality were also found in Ge‐incorporated CZTSSe solar cells.^[^ ^16^, ^18^ ^]^ Undoubtedly, Ge incorporation has emerged as an indispensable process, demonstrating its essential role in enhancing kesterite performance. Therefore, unveiling the underline mechanism of Ge incorporation holds immense significance in exploiting the full potential of this step‐changing approach that could lead to transformative advancements in kesterite technology.

In this work, we reveal the role of Ge in CZTSSe as a function of the amount of Ge incorporated and associated underlying mechanism for performance improvement by examining the selenization process at different annealing time points. For this purpose, we prepared samples with varied Ge thickness deposited on the pre‐alloyed Cu‐Zn‐Sn precursor. A range of micro‐to‐atomic‐scale characterizations including scanning transmission electron microscopy (STEM), energy dispersive X‐ray spectroscopy (EDS), high resolution transmission electron microscopy (HRTEM), X‐ray diffraction (XRD), cathodoluminescence (CL), time of flight secondary ion mass spectrometry (TOF‐SIMS) and atom probe tomography (APT) were implemented to track elements distribution and identify phase transitions as a result of Ge incorporation. Our research findings demonstrate that the inclusion of Ge in the kesterite structure results in a delay in the kesterite formation process and such delay increases with excess Ge amount. This delay can be attributed to an early formed ZnSe_x_ phase at the top region of the film which is able to suppress Zn loss and promotes the formation of Cu‐Sn alloy.^[^ ^19^ ^]^ Consequently, the formation of the ternary Cu‐Sn‐Se compound precedes kesterite formation. Furthermore, the introduction of the optimal amount of Ge improves crystallization and promotes the growth of large grains, reducing detrimental horizontal grain boundaries observed frequently in Ge‐free CZTSSe solar cells. The improved understanding of the role of Ge in kesterite CZTSSe could be potentially extended to other compound materials based solar cells with similar sequential deposition and heating strategies to explore the potential performance improvement by such extrinsic element additive.

## 2 Results and Discussion

The addition of Ge in small amounts has been shown to enhance the performance of kesterite solar cells by promoting the favorable reaction pathway of CZTSSe, reducing Sn‐related defects and improving CZTSSe grain morphology, as reported in previous studies.^[^ ^11^, ^12^, ^14^, ^15^, ^16^ ^]^ Consistent with these findings, our study also found that incorporating a trace amount of Ge in kesterite solar cells resulted in increased efficiency, with an average efficiency of 10.69% for samples incorporating a 5 nm Ge layer, compared to 8.06% for Ge‐free reference samples as shown in Table S1 (Supporting Information). However, excess Ge incorporation (a 50 nm layer) had a detrimental impact on the performance of device arising from deep midgap defects.^[^ ^20^ ^]^

In this study, we selected Ge nanolayers of 0 nm, 5 nm as well as 50 nm to gain insights into the impact of Ge on the kesterite formation pathway. To prepare these samples, the stacked Cu/Zn/Sn metallic precursors underwent a pre‐alloying process at 300 °C for 60 min. Then, Ge layers with either 5 nm or 50 nm thickness were sputtered on top of these pre‐alloyed Cu‐Zn‐Sn precursors. A rapid thermal selenization process was then applied to form kesterite thin films. The selenization consisted of a 150 s heating process to reach 520 °C from room temperature, then holding at 520 °C for 7 mins 30 s to ensure sufficient selenization and then cooling naturally to ambient temperature.

To investigate the effect of Ge during kesterite growth, the reactive selenization process was interrupted at four different time points (after just reaching 520 °C, 520 °C after 1 min, 520 °C after 3 min 45 s and 520 °C after 7 mins 30s), denoted as t1, t2, t3, t4, respectively, as shown in Figure S1 (Supporting Information). The entire selenization duration is set at 10 min, starting from room temperature to the end of the process. t1: This point is selected as it marks the first moment when the temperature reaches 520 °C. It's a critical threshold for initiating significant chemical and structural changes essential for the formation of the CZTSSe phase. t2: Chosen to investigate the early stage of phase evolution. This time point provides insights into the initial reactions and transformations occurring as the temperature rises. t3: Represents the midpoint between t1 and t4. This time point is crucial for understanding the progress of the phase transformation process at an intermediate stage. t4: Selected as it corresponds to the conclusion of the selenization process. This final point helps in assessing the end result of the selenization, including the final phase composition and structure. Characterization was conducted on samples with 5 nm and 50 nm Ge layers and without Ge at these different selenization time points. These three series of samples were defined as Ge5, Ge50 and Ref, respectively. The details of the characterization methods conducted on each sample are summarized in Table S2 (Supporting Information), and the role of Ge in the growth of kesterite is discussed in the following Sections 2.1‐2.3.

### 2.1 Delayed Kesterite Formation in the Selenization Process

Analyses of all samples at different selenization time points were conducted by STEM‐EDS and XRD to investigate elemental distributions and phase evolution. The results demonstrated the introduced Ge delayed kesterite formation by forming a ZnSe on the top of precursor and promoting Cu‐Sn‐Se phase formation, which will further alter the kesterite formation mechanism.

#### 2.1.1 Ge‐Free Sample

**Figure 1:**
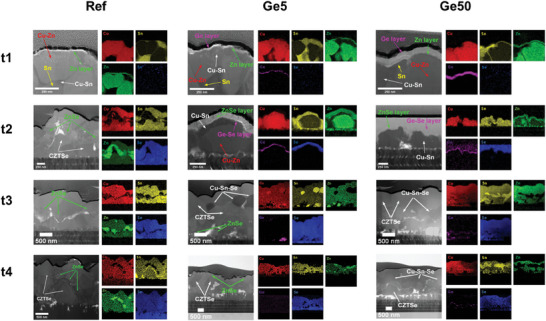
Cross‐sectional STEM and EDS mappings of the CZTSSe absorbers with different thickness of Ge layers deposited on the precursors. The selenization process was interrupted at different times t1, t2, t3, and t4, as marked in the temperature profile (Figure S1, Supporting Information).

At t1, the absence of binary and ternary selenide phases in Ref sample (**Figure** **1**) indicates that the selenization has not yet commenced upon reaching a temperature of 520 °C. The presence of Cu‐Zn alloy was observed, with minimal formation of Cu‐Sn alloy. Additionally, Figure S2a (Supporting Information) shows that Cu‐Zn alloy was prevalent with a partial amount of Cu alloyed with Sn, leading to the formation of Cu‐Sn alloy, while residual Sn was located near Mo and filled the absorber. These observations suggest that, during the pre‐alloying process, Cu exhibited a stronger tendency to form alloys with Zn rather than Sn, likely due to the lower activation energy associated with Cu‐Zn alloy formation compared to that of Cu‐Sn alloys.^[^ ^21^, ^22^ ^]^ It can also be noted that, as shown in Figure 1, a thin Zn layer was found on the top of the metal alloy at t1, indicating Zn evaporation occurred with temperature increasing since Zn can volatilise at 250 °C and redeposit on the surface.^[^ ^23^ ^]^ The formation of the kesterite phase in the Ref sample commenced approximately at t2, as indicated by the overlapping mapping signals of Se with Cu, Zn, and Sn observed in Figure 1. This suggests that the time window for the conversion of kesterite from the alloyed phase is relatively narrow, spanning only ≈1 min, which poses limitations on the regulation of intermediate phases. The EDS mapping results at t3 and t4 further confirmed the presence of the kesterite and ZnSe phases, as depicted in Figure 1, implying most XRD peaks at t3 and t4 belong to kesterite or ZnSe phases as XRD is not reliable in distinguishing CZTSe and secondary phase or ZnSe due to their overlapping XRD patterns.^[^ ^24^ ^]^

#### 2.1.2 Sample with 5 nm Ge

The kesterite formation pathway started to change when Ge was introduced. The STEM‐EDS mapping (Figure 1) results for Ge5 sample showed that, at t1, Cu mixed well with Zn as Cu‐Zn alloy, partial Sn alloyed with excess Cu and unreacted Sn exist as metallic phase. A thin Zn layer was also found on the top of the absorber, covered by an ultrathin Ge nanolayer, similar to the reference sample without Ge. The only difference here is the existence of an additional Ge overlying the Zn layer. CuZn and Cu_5_Zn_8_ peaks were identified in XRD for the Ge5 absorber (Figure S3a, Supporting Information), supporting the observation in EDS mapping results at t1 (Figure 1). However, the reaction route changed at the following time points. Unlike Ref, a ZnSe_x_ layer located on top of the absorber and a Ge_x_Se_y_ layer existed between ZnSe_x_ and absorber at t2, as depicted in Figure 1.

However, CZTSSe was not detected in the EDS mapping at this stage. This suggests that kesterite conversion from alloyed phases in the Ge5 sample was delayed, resulting in a higher degree of decomposition of the Cu‐Zn alloy and the subsequent formation of ZnSe_x_. This was likely due to a longer exposure to Se vapor at a higher temperature. Thermodynamic calculation indicates that the formation of ZnSe is more favorable over Cu_2_Se due to larger negative Gibbs free energy for ZnSe when selenium reacts with Cu‐Zn alloy.^[^ ^25^ ^]^ As a result, Cu from decomposed Cu‐Zn near the upper region exhibited a tendency to alloy with Sn, forming Cu‐Sn alloy (as shown in Figure 1), which is in line with the findings from XRD analysis (Figure S3b, Supporting Information). Additionally, a very thin Ge layer with overlapping signal of Se was detected at the same depth as the ZnSe layer at t2, while no Ge diffusion into the precursor was observed in Figure 1. Note that surface scanning electron microscopy (SEM) image also demonstrated a liquid‐spread morphology, as demonstrated in Figure S4b (Supporting Information), corresponding to the observation of the Ge_x_Se_y_ phase layer forming on top of the precursor in Figure 1e. Matrix elements including Cu, Zn, Sn and Se were well mixed in most regions at t3 from EDS mapping (Figure 1), implying kesterite phase started forming between t2 and t3 with a Cu‐Sn‐Se phase also observed. At t4, kesterite and ZnSe were found from EDS mapping and no Cu‐Sn‐Se phase was identified. This proved that the introduced Ge delays the kesterite formation in the selenization process by forming a thick ZnSe layer on the top. Meanwhile, Cu‐Sn alloy started to form due to Cu‐Zn alloy decomposition, then forming a ternary Cu‐Sn‐Se phase near the front interface, effecting the subsequent selenization process to form kesterite. A similar change in kesterite formation mechanism has been reported by IREC^[^ ^14^ ^]^ with Ge layer deposited below the metallic stacked precursor without pre‐alloy treatment. In their case, the introduced Ge drastically modified the reaction pathway from a binary selenide reaction without Ge involved to a ternary selenide pathway with Ge incorporation, achieved by inhibiting fast Cu‐out diffusion and Sn loss and thus promoting Cu‐Sn‐Se ternary phase formation. Therefore, regardless of the original position of Ge nanolayer, introduced Ge nanolayer is capable to alter the kesterite formation pathway by promoting the Cu‐Sn phase formation and subsequently effecting Cu‐Sn‐Se ternary phase formation.

#### 2.1.3 Sample with 50 nm Ge

The scenario for the Ge50 sample was similar to the Ge5 sample. At t1, a thicker Ge nanolayer on the surface as well as a Zn layer underneath the Ge layer (Figure 1) were detected in EDS mapping. At t2, the whole absorber was covered by a thick and flat Ge‐Se phase confirmed by localised EDS mapping (Figure S5, Supporting Information), which explains the unexpected smooth morphology shown in Figure S4c (Supporting Information). A ZnSe_x_ layer was detected between thick Ge‐Se phase layer and precursor at t2. Ge signal could be both detected in EDS mapping at t3 and t4, along the edge of voids and cracks. The Ge50 sample had the same XRD diffraction peaks as the Ge5 sample at t1 as shown in Figure S3a (Supporting Information). The EDS mapping analysis of the Ge50 sample at time points t3 but also t4 revealed the presence of the Cu‐Sn‐Se phase, indicating that an excess amount of Ge could contribute to the increased formation of Cu‐Sn alloys and the subsequent formation of excess Cu‐Sn‐Se. In other words, the delay in the selenization process due to Ge incorporation is more pronounced in the Ge50 sample. The thick liquid Ge‐Se phase layer prevented heat and mass transfer into the underlying precursor and thus reduced the effective selenization duration, affecting the subsequent kesterite formation process.

For the samples with the Ge nanolayer deposition, regardless of the Ge thickness, the kesterite formation was different from the Ref (Ge‐free) sample. First, the introduced Ge nanolayer effectively delayed the conversion into the kesterite phase by forming a ZnSe_x_ layer on the top of metal alloys at the early stage of selenization and the ZnSe_x_ layer can suppress Zn loss due to Zn metal volatility. The tendency of forming ZnSe over Cu_2_Se upon the reaction with Se and Cu‐Zn alloy promote the decomposition of Cu‐Zn alloy and the formation of Cu‐Sn alloy, resulting the subsequent formation of a Cu‐Sn‐Se phase. Instead of the narrow kesterite conversion window for Ge‐free samples, we observed that the processing window can be widened by forming of a ternary Cu‐Sn‐Se phase to promote the formation of CZTSSe kesterite.^[^ ^14^ ^]^ In addition, the delay effect is dependent on the thickness of Ge; thicker Ge can form more liquid phase Ge_x_Se_y_ which regards heat/mass transfer into precursor, slows down the temperature rise and shortens the effective selenization duration, which is not conducive to kesterite formation. This hypothesis regarding the impact of the Ge_x_Se_y_ phase has been substantiated by observing variations in the thickness of the MoSe2 layer.

**Figure 2:**
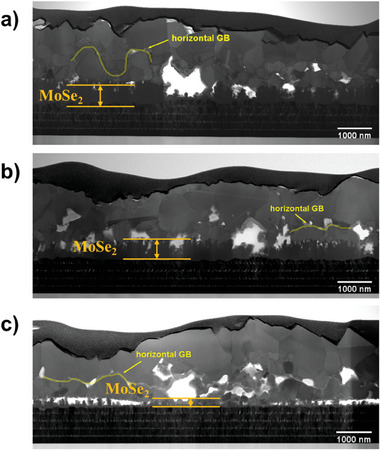
Cross‐section STEM images of absorbers at t4: a) Ref; b) Ge5; c) Ge50.

A comparable thickness of an interfacial MoSe_2_ layer between absorber and Mo layer was also observed in Ref and Ge5 absorbers as demonstrated in **Figure** **2**a and Figure 2b. An ultra‐thick MoSe_2_ layer is considered detrimental to device performance by increasing the series resistance, as it will decrease the thickness of the Mo substrate and influence the electrical contact of the CZTSSe absorber and Mo layer.^[^ ^26^ ^]^ A reverse correlation between MoSe_2_ thickness and device performance was observed in kesterite^[^ ^26^ ^]^ and Cu(In,Ga)Se_2_.^[^ ^27^ ^]^ The thickness of MoSe_2_ reduced dramatically in the Ge50 absorber as shown in Figure 2c.^[^ ^28^ ^]^ Typically, the significant growth of MoSe_2_ grows is observed above 450 °C.^[^ ^29^ ^]^ In case of Ge50 sample, the volatilisation of a dense, liquid Ge_x_Se_y_, which forms between temperatures t1 and t2, hinders the transfer of heat and mass into the absorber. Consequently, spending a shorter annealing duration above 450 °C during the selenization process could explain the formation of a thinner MoSe2 layer in the Ge50 sample due to the delaying effect.

### 2.2 Reduced Detrimental “Horizontal” Grain Boundaries

The effect of Ge incorporation on kesterite morphology was further investigated by STEM‐EDS, HRTEM and CL measurement to investigate the film quality in more detail. The cross‐section STEM image (Figure 2a) shows formation of horizontal grain boundaries between the upper layer, consisting of large grains, and the bottom layer, composed of small grains within all absorbers. The composition of this type of grain boundary was further analyzed by HRTEM and EDS mapping images in Figure S6a (Supporting Information). It shows that nanocrystals with nearly round shape comprised of Sn and O exist at horizontal grain boundaries and distribute unevenly with space between them. The SnO_x_ nanocrystals with high band gap at the grain boundaries can be expected to act as a current barrier preventing carrier transport.^[^ ^30^ ^]^ The large voids near the rear area may originate from the volatile phase SnSe, which caused uncontrolled Sn‐loss during the reaction.^[^ ^31^ ^]^ Sn loss due to volatile phase evaporation is commonly observed in kesterite CZTS solar cells.^[^ ^32^ ^]^

**Figure 3:**
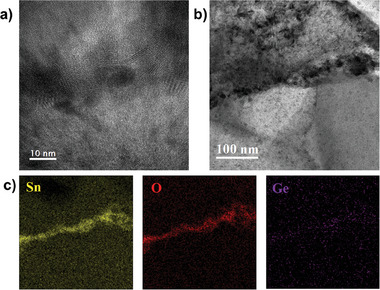
a), HRTEM image showed crystallised nano clusters at horizontal grain boundaries of Ge5 at t4. b), TEM image of a horizontal boundary of Ge5 at t4. c), Corresponding EDS mapping images of the horizontal grain boundary in (b).

The grain morphology was modified in Ge5 and Ge50 samples compared to the reference sample. The Ge5 and Ge50 absorbers consisted of much larger grains on the upper layer with smaller grains on the bottom (Figure 2b,c). Horizontal grain boundaries including SnO_x_ and GeO_x_ nano‐inclusions were observed between large grains and small grains in both the Ge5 and Ge50 absorber, supported by TEM and EDS mapping shown in **Figures** **3** and S6b (Supporting Information), respectively. Compared with near vertical grain boundaries, which did not show obvious fluctuations in the elemental distribution (Figure S7, Supporting Information), horizontal grain boundaries exhibited pronounced enrichment of Sn, O and Ge, according to EDS mapping images shown in Figures 3c and S6b (Supporting Information) as well as a line scan elemental distribution shown in Figure S7d (Supporting Information). GeO_x_ inclusion with the order of 1–10 nm diameter has been reported at grain boundaries in the previous study.^[^ ^12^ ^]^ However, GeO_x_ was also found embedded within the large grain and exhibited Sn‐neutral and separated with SnO_x_, which are absent in our results as demonstrated in Figure 3c. In our case, such differential could be raised by the pre‐alloying process prior to the selenization, which may influence the distribution and interaction Ge within the material, leading to distinct results. Further investigations may require probing the pre‐alloying process's effects on the GeOx distribution. A HRTEM image (Figure 3a) indicates crystallized nanoclusters formed at the horizontal grain boundaries. It is reasonable to conclude that the horizontal grain boundaries were porous, not compact, capturing more oxygen into the boundaries. The oxygen may originate from exposure of as‐deposited precursor or pre‐alloyed precursor to air before selenization. This assumption is further supported by detected oxygen signals at t1 for all three samples, as illustrated in Figure S8a (Supporting Information). From STEM‐EDS images in Figure 1, it can be observed that the horizontal grain boundaries were formed after the selenization process started for all the three samples, i.e., t2 for Ref while t3 for Ge5 and Ge50. We suspect that volatile Sn‐related and Ge‐related phases evaporate during selenization and grain growth, combining with oxygen and resulting in the formation of nano clusters at horizontal grain boundaries, separating the large‐grained upper layer and the smaller‐grained bottom layer. The delayed selenization process induced by Ge may enable sufficient mass transfer and element interdiffusion, resulting in large grains on the upper layer.^[^ ^33^ ^]^

**Figure 4:**
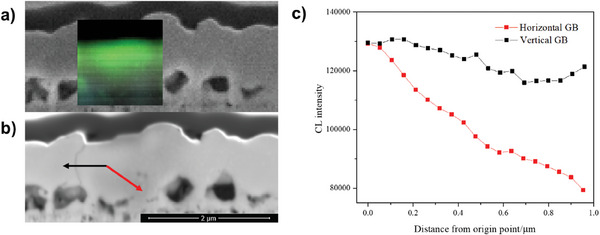
a), CL map acquired at 10 keV with 20s exposure time from a Plasma FIB‐prepared cross‐sectional sample of Ge5 at t4., CL map. b), Backscatter image of a Plasma FIB‐prepared cross‐sectional sample of Ge5 at t4. The black arrow marks the near vertical grain boundary and red arrow marks the horizontal grain boundary. c), Extracted CL intensity across the two grain boundaries as marked in (b).

To better understand these horizontal grain boundaries containing Sn and Ge enrichment inclusions, spatially and spectrally resolved CL was employed on the Ge5 sample to investigate their effect on electrical properties. CL line scans cross two representative grain boundaries in the Ge5 sample at t4, i.e., near vertical grain boundaries and horizontal grain boundaries are shown in **Figure** **4**. Apparently much lower CL intensity was observed at the horizontal grain boundary, indicating that the horizontal grain boundary had much larger non‐radiative recombination velocity than that near the vertical grain boundary.^[^ ^10^ ^]^ The length of horizontal grain boundaries in the Ge5 absorber reduced significantly compared to the Ref absorber, enhancing carrier transport. Also, the small grains at the bottom had lower CL intensity than large grains on the top indicating that small grains had more defects and shorter lifetimes. Meanwhile, the larger grain size reduced the length of near‐vertical grain boundaries. High effective recombination velocity at grain boundaries has been reported as one of the main limiting factors for kesterite solar cells.^[^ ^10^ ^]^ In conclusion, detrimental horizontal grain boundaries with larger non‐radiative recombination velocity than near vertical grain boundaries were confirmed by CL measurement, also larger grains on the top can reduce the length of horizontal grain boundaries, further enhancing carrier transport and inhibiting recombination, which is beneficial to device performance.

### 2.3 Ge Footprint Tracking

To further elucidate the Ge role in kesterite growth as well as evaluate the exact amount of Ge in the absorber at the final stage, it is vital to determine the Ge footprint during the selenization process. According to EDS mapping results presented in Figure 1, Ge was initially observed to be localized overlying the precursor layer, where subsequent reaction with Se resulted in the formation of a liquid phase Ge_x_Se_y_, which served as a carrier for incorporation of Ge into the kesterite bulk. However, there was no evidence derived from EDS results to prove the presence of Ge in the grain interior (GI) in the final film. We found that Ge was primarily detected at structural defects such as near the voids and at horizontal grain boundaries, presenting in a Sn‐Ge oxide form.

With the increasing Ge thickness, the XRD diffraction peak assigned to the kesterite is slightly shifted to higher angles (Figure S9, Supporting Information), indicating the partial substitution of Sn components in the kesterite lattice with Ge.^[^ ^34^ ^]^ A SIMS depth profile with the same level signal of Mo (Figure S10, Supporting Information) shows a slightly higher Ge intensity in the Ge50 sample than in the Ge5 sample. In addition, Na was found to be attracted to the surface area when more Ge was introduced, as evidenced by not only the higher Na intensity in Ge50 than Ge5 sample, but also the shift of the intensity peak toward the surface direction. Ge was found to have strong interaction with Na since Na is reported to diffuse toward the absorber surface from soda lime glass when thick Ge nanolayer is deposited on top of precursors.^[^ ^35^ ^]^

**Figure 5:**
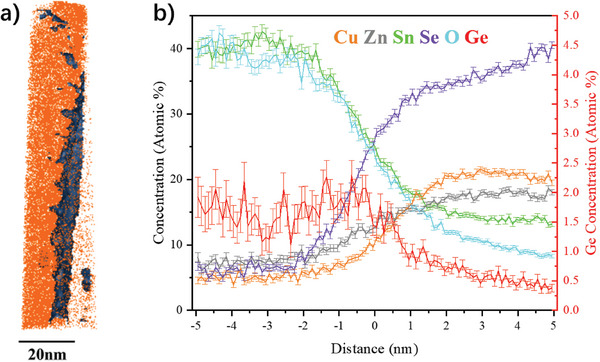
a) Three‐dimension maps of Cu (orange) atoms from a dataset of Ge5 at t4. The dark blue isoconcentration surface mark 13 at. % Cu as horizontal grain boundary. b) Proxigram of the 13 at. % Cu isoconcentration surface. Distance zero corresponds to the position of isosurface, the positive distance to the grain interior and the negative distance to the horizontal grain boundary.

APT can provide 3D compositional maps with atomic spatial resolution, and is a powerful tool in mapping the trace elements.^[^ ^36^ ^]^ APT was applied to gain a more comprehensive understanding of the remaining Ge distribution within the grain interior. A bulk concentration profile shows that the Ge concentrations in GI were 0.097% and 0.492% for Ge5 and Ge50, respectively. (Table S3, Supporting Information). Considering that the quantity of Ge added in Ge50 was 10 times that in Ge5, the Ge concentration in the final stage of Ge50 sample was only ≈5 times that in Ge5 sample. It indicated that there might be a limitation to introducing Ge into kesterite by introducing an ultrathin Ge nanolayer prior to the selenization process. A significant portion of Ge was evaporated as selenide phase rather than being incorporated into grain interior. Additionally, a presence of a thick liquid Ge_x_Se_y_ phase on top of metal alloys may affect the heat transfer to the absorber and mass transport thermodynamically, consequently restricting the capability of Ge incorporation into the crystal lattice.^[^ ^37^ ^]^ In addition to detect Ge and the matrix elements concentration in the grain interior, it was also necessary to obtain the exact composition at the horizontal grain boundaries. Due to the void structure at the bottom of the sample, a specially designed tip preparation method was used to interrogate horizontal grain boundaries as much as possible while decreasing the possibility of tip fracture. A significant Sn and O enrichment and Cu, Zn, Se depletion was observed in one APT tip for the Ge5 sample. As discussed above and confirmed by EDS mapping, this indicated a horizontal grain boundary was successfully captured by this tip specimen (Figure S11, Supporting Information). A proxigram^[^ ^38^ ^]^ using 13% Cu isoconcentration surface to define the horizontal grain boundary showed that the concentration of Ge increased from 0.5% in the grain interior to ≈2% in the horizontal grain boundary (**Figure** **5**), demonstrating an increasing Ge concentration gradient from grain interior to horizontal grain boundaries.

Through comprehensive micro‐to‐atomic characterisation, it has been determined that during the selenization process, Ge follows a specific pathway. Initially, Ge combines with Se, forming a liquid phase Ge_x_Se_y_, which then diffuses toward the bottom of the film. However, only a limited amount of Ge enters the grain interior to substitute Sn, while a significant portion of Ge forms oxide precipitates at the grain boundaries in conjunction with tin oxide, serving as a barrier to current flow. It is worth noting that despite the presence of residual Ge within the grain interior and horizontal grain boundaries, a substantial portion of Ge evaporates in the form of volatile selenide.

## 3 Conclusion

In this work, we significantly advance the understanding of the role of Ge in pre‐alloyed CZTSSe solar cells, fabricated using metallic stacked Cu/Zn/Sn precursors, gaining valuable insights into the selenization process through comprehensive micro‐to‐atomic characterisations. It is revealed that, by forming a ZnSe layer on the precursor, a Ge nanolayer incorporated on the precursor can inhibit Zn loss occurred at early stage and delay the kesterite formation in the selenization process till higher temperature. This delay is essential as it promotes the formation of Cu‐Sn alloys, transforming the kesterite formation from a binary selenide reaction to a ternary Cu‐Sn‐Se phase reaction. Furthermore, appropriate Ge incorporation may improve crystallinity and grain morphology by enlarging grain size and reducing harmful horizontal grain boundaries. However, excessive Ge incorporation can have adverse effects, leading to a more severe delay in the selenization process, significantly affecting heat and mass transfer and subsequent kesterite formation. The pathway of Ge in the formation of kesterite was determined to be that a limited amount of Ge can partially substitute Sn in the grain interior and some precipitate out as GeO_x_ at horizontal grain boundaries, while most of Ge evaporates out during the selenization process. The insight inspired by this work will deepen the understanding of the role of Ge or other extrinsic elements during the selenization process in CZTSSe kesterite solar cells, which may be able to be extended to other chalcogenide solar cells and provide a strategy to improve device performance and grain growth.

## 4 Experimental Section

### Precursor and Absorber Preparation

The metallic precursor thin films were prepared by sputtering metallic targets in sequence of Zn, Sn, and Cu on Mo‐coated SLG substrates by means of a DC sputtering technique. The sputtering conditions such as power density, working pressure, and deposition time for each metallic layer were as follows: Cu (0.68 W cm^−2^,8 mTorr, 2812 s), Sn (0.68 W cm^−2^, 8 mTorr, 2015 s), and Zn (0.68 W cm^−2^, 8 mTorr, 1806 s). A necessary prealloying process was applied.^[^ ^28^, ^39^ ^]^ The precursor thin films were annealed under an Ar atmosphere (1 atm) at 300 °C for 60 min to form the Cu–Zn and Cu–Sn alloys, and then cooling down to room temperature naturally. The ramping rate of the process was 10 °C min^−1^. 5 nm and 50 nm Ge layers were deposited by a DC sputtering upon the prealloyed Cu‐Sn‐Zn precursor with a deposition rate of 0.57 Å s^−1^.^[^ ^40^, ^41^ ^]^ After precursor preparation, S (0.002 g) and Se (0.198 g) powders and precursor were placed in a graphite box in a closed isothermal chamber system and annealed at 520 °C for 10 min for the growth of CZTSSe‐Ge. A CdS buffer layer, ≈20–25 nm thick, was deposited via chemical bath deposition (CBD). Following this, the intrinsic ZnO (i‐ZnO) and aluminium‐doped ZnO (AZO) layers were deposited using the RF magnetron sputtering process. 1000 nm Al grids were then applied to the AZO layer using a mask in the sputtering process. The active area of the kesterite thin film solar cells, defined by mechanical scribing, was 0.3 cm^2^.

### Characterisation Methods

The TEM specimens were prepared using a focused ion beam with a microsampling system (FEI xT Nova NanoLab200). The microstructure and compositional profiles were measured using a JEOL F200 (200 kV) cold feld‐emission gun (FEG) scanning transmission electron microscope equipped with a windowless silicon drift energy dispersive x‐ray (EDX) detector. The element depth profiles were recorded by SIMS (TOF‐SIMS^5^), analysed in positive polarity by Bi_3_^+^ at 30 KeV and sputtered by Cs^+^ beam of 2000 eV in MCs mode. X‐ray diffraction (PANalytical Empyrean thin film XRD) was used for detecting the structure of the films under 45 kV voltage, 40 mA current using Cu Kα radiation (λ = 1.54Å). Surface morphological analysis was performed using a SEM (NanoSEM 450). The cross‐section sample for CL was prepared by a plasma FIB (Thermo Fisher Helios G4 PFIB) and a final ion beam polish was applied by Fischione NanoMill 1040 to ensure a clean and fresh surface. CL analysis was conducted using Delmic SPARC spectral cathodoluminescence system coupled to an FEI Nova Nano SEM 450 field‐emission SEM. A 10 kV and 4.0 spot size parameter combination in SEM is chosen for measurements. Considering that the bandgap of CZTSSe, an IR detector was used and a 20s exposure time was selected to ensure enough signal and prevent software crash during too long measurements which happened occasionally. APT needle specimens were lifted out in a in a focused‐ion beam/scanning electron microscope (Helios G4 PFIB UXe) and the tips direction were parallel to the substrates that was achieved by making a right triangle shape cantilever at the beginning, then the cantilever was rotated for 90 degrees twice using the manipulator and was welded to the post for further annular milling. APT experiment was performed in a Local Electrode Atom Probe (LEAP 4000X Si, CAMECA) at a pulse frequency of 200 kHz, a stage temperature of 50 K and a laser pulsing energy of 100 pJ. APT data analysis was performed in the commercial software APSuite (Version 6.1.0.29, CAMECA).

## Conflict of Interest

The authors declare no conflict of interest.

## Supporting information

Supporting Information

## Data Availability

The data that support the findings of this study are available from the corresponding author upon reasonable request.
