# Supplementary material for: Unveiling the Role of Ge in CZTSSe Solar Cells by Advanced Micro‐To‐Atom Scale Characterizations
Source: Adv Sci (Weinh). 2024 Feb 11;11(15):2305938. doi: 10.1002/advs.202305938 (PMC11022695; doi:10.1002/advs.202305938)
Supplement: Supplementary file 1 — Supporting Information [file ADVS-11-2305938-s001.pdf]

ADVANCED SCIENCE
Open Access

Supporting Information

for *Adv. Sci.*, DOI 10.1002/advs.202305938

Unveiling the Role of Ge in CZTSSe Solar Cells by Advanced Micro-To-Atom Scale Characterizations

Jialin Cong, Mingrui He, Jun Sung Jang, Jialiang Huang, Karen Privat, Yi-Sheng Chen, Jianjun Li, Limei Yang, Martin A. Green, Jin Hyeok Kim*, Julie M. Cairney* and Xiaojing Hao**

Supplementary Information

Jialin Cong[1], Mingrui He[1], Jun Sung Jang[2], Jialiang Huang[1*], Karen Privat[3], Yi-Sheng Chen[4], Jianjun Li[1], Limei Yang[5], Martin A. Green[1], Jinhyeok Kim[3*], Julie Cairney[4*], Xiaojing Hao[1*]

1. Australian Centre for Advanced Photovoltaics, School of Photovoltaic and Renewable Energy Engineering, University of New South Wales, Sydney, New South Wales, Australia
2. Department of Materials Science and Engineering, Chonnam National University, Gwangju, Republic of Korea
3. Electron Microscope Unit, Mark Wainwright Analytical Centre, University of New South Wales, Sydney, New South Wales, Australia
4. Australian Centre for Microscopy and Microanalysis (ACMM), The University of Sydney, Sydney, New South Wales, Australia
5. School of Civil and Environmental Engineering, University of Technology Sydney, Sydney, New South Wales, Australia

* Corresponding author: jialiang.huang@unsw.edu.au, jinhyeok@chonnam.ac.kr, julie.cairney@sydeney.edu.au, xj.hao@unsw.edu.au

Table S1. The average device characteristics of the CZTSSe and CZTSSe-Ge solar cells.

Device	V_{oc} [mV]	J_{sc} [mA cm $^{-2}$]	Fill Factor [%]	Efficiency [%]
CZTSSe-ref	472.58	34.04	50.15	8.06
CZTSSe-Ge (5 nm)	517.82	33.57	61.53	10.69

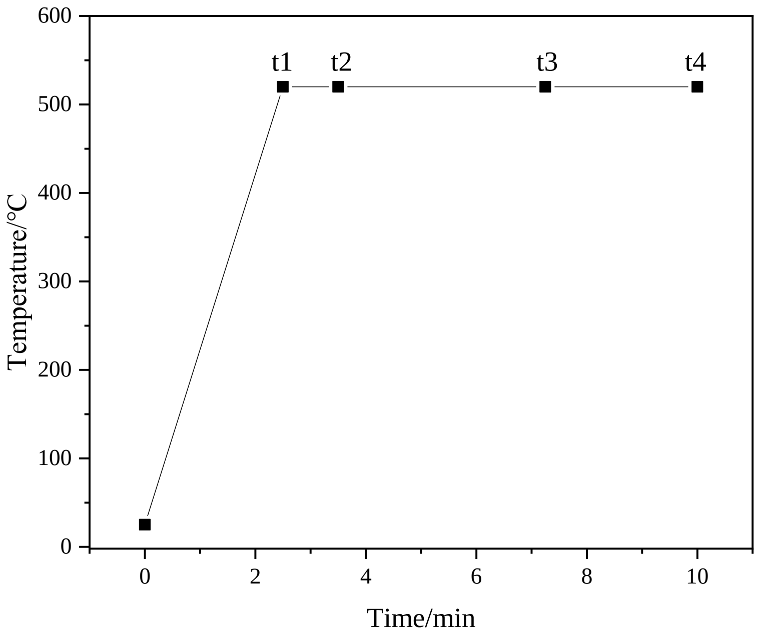

Figure S1. The selenization process was interrupted after different times t1 (just reaching at 520 °C), t2 (520 °C after 1 min), t3 (520 °C after 3 min 45 s), t4 (520 °C after 7 mins 30s), as marked in temperature profile.

Table S2. Summary of characterization methods conducted on Ref, Ge5 deposition and Ge50 deposition absorbers.

Sample	Time point	STEM-EDS	XRD	SIMS	SEM	APT	HRTEM	CL
**Ref**	t1	✓	✓		✓			
	t2	✓	✓		✓			
	t3	✓	✓		✓			
	t4	✓	✓	✓	✓		✓	
**Ge5**	t1	✓	✓		✓			
	t2	✓	✓		✓			
	t3	✓	✓		✓			
	t4	✓	✓	✓	✓	✓	✓	✓
**Ge50**	t1	✓	✓		✓			
	t2	✓	✓		✓			
	t3	✓	✓		✓			
	t4	✓	✓	✓	✓	✓	✓	

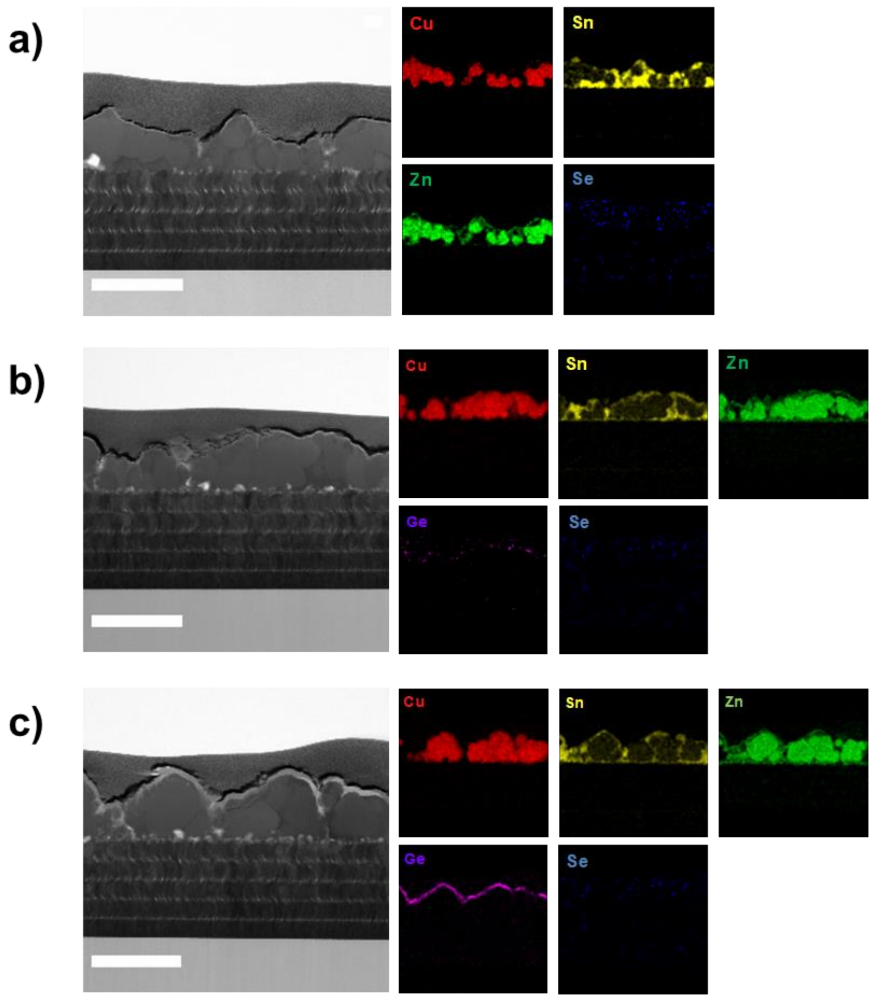

Figure S2. STEM and EDS mapping images at t1, a) Ref; b) Ge 5; c) Ge 50, all images share same scale bar which represent 1 micron.

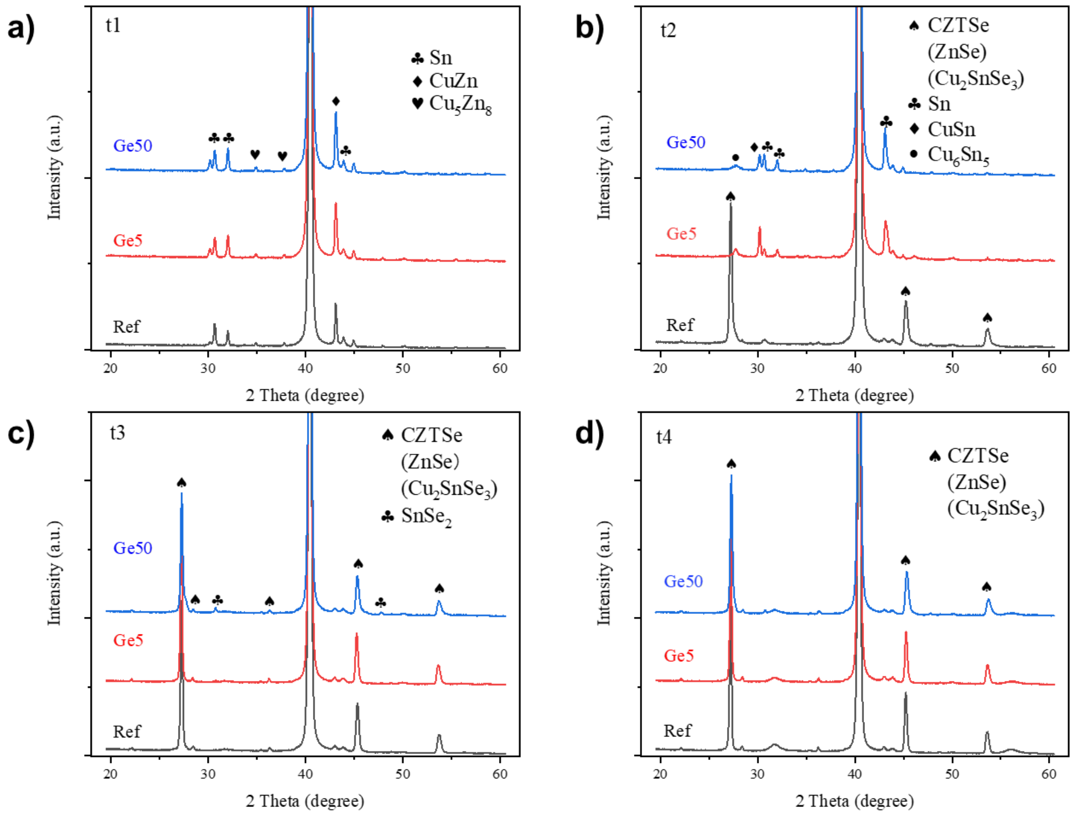

Figure S3. XRD diffractograms of three samples, a) t1; b) t2; c) t3; d) t4.

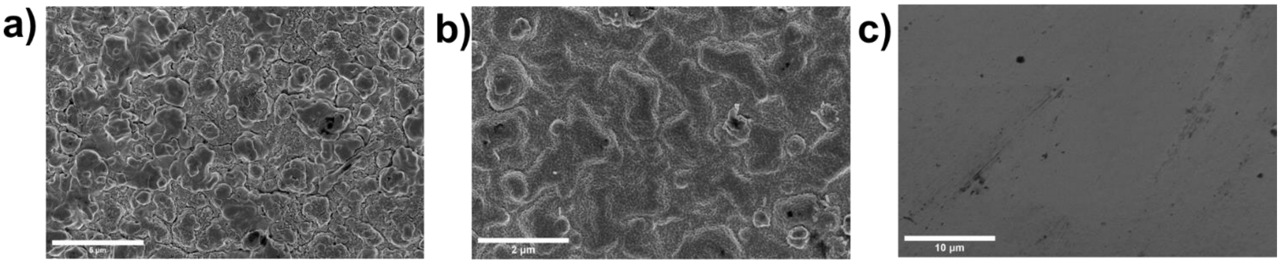

Figure S4. Surface SEM images at t2, a) Ref; b) Ge5; c) Ge50.

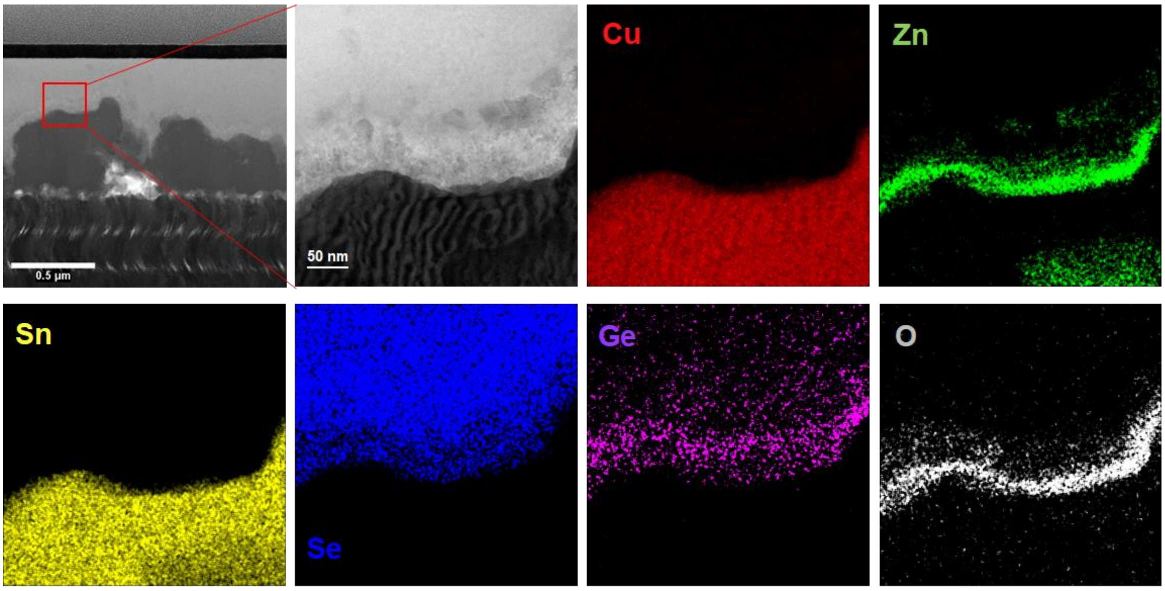

Figure S5. Localized EDS mapping for Ge50 at t2.

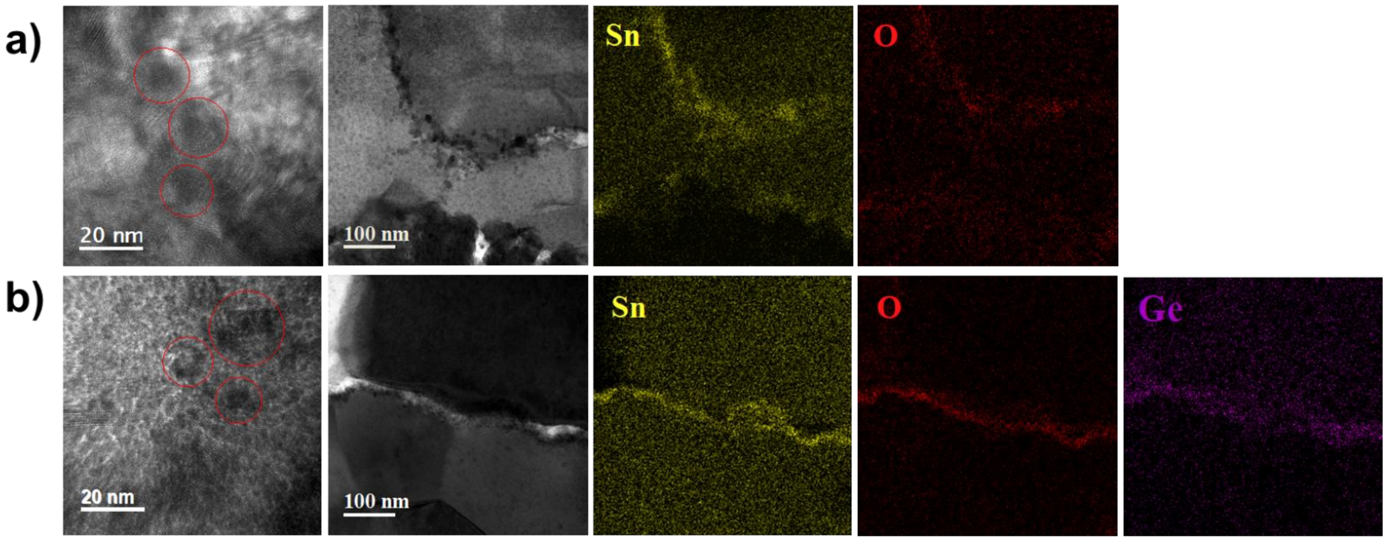

Figure S6. High resolution TEM (HRTEM) images and EDS mapping images, a) ref and b) Ge50 and red circles indicate the nanocrystals.

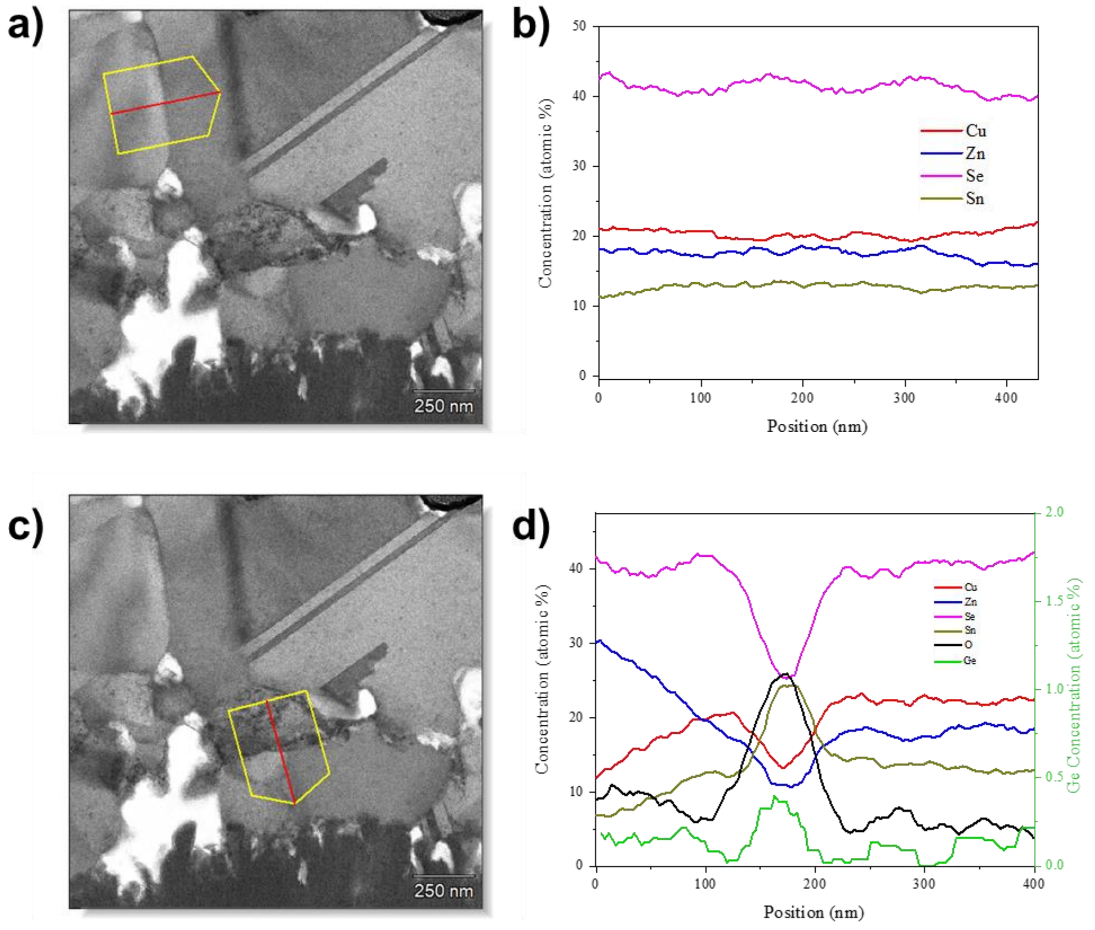

Figure S7. Bright-field STEM images of cross-sectional Ge5 samples, a) near-vertical grain boundary; c) horizontal grain boundary. The EDS line scan profiles across grain boundary at the corresponding position labeled in STEM images, b) near-vertical grain boundary; d) horizontal grain boundary.

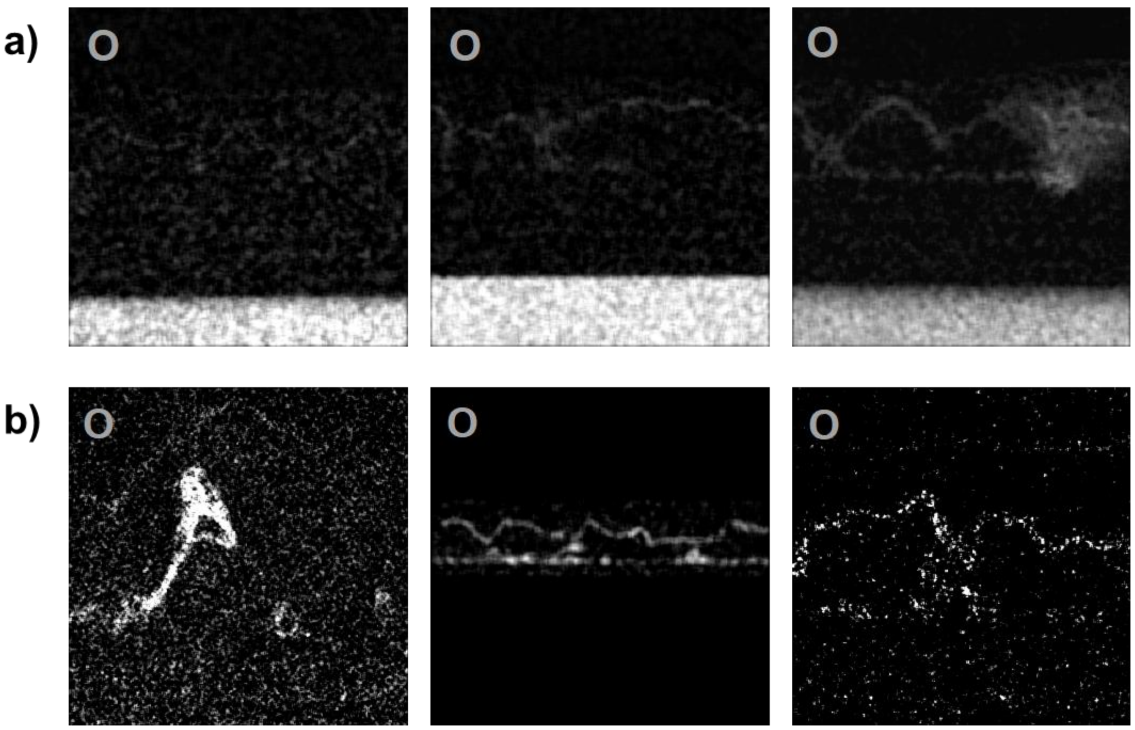

Figure S8. Oxygen signals derived from the EDS mapping at t1 (a) and t2 (b), from left to right are Ref, Ge5 and Ge50, respectively.

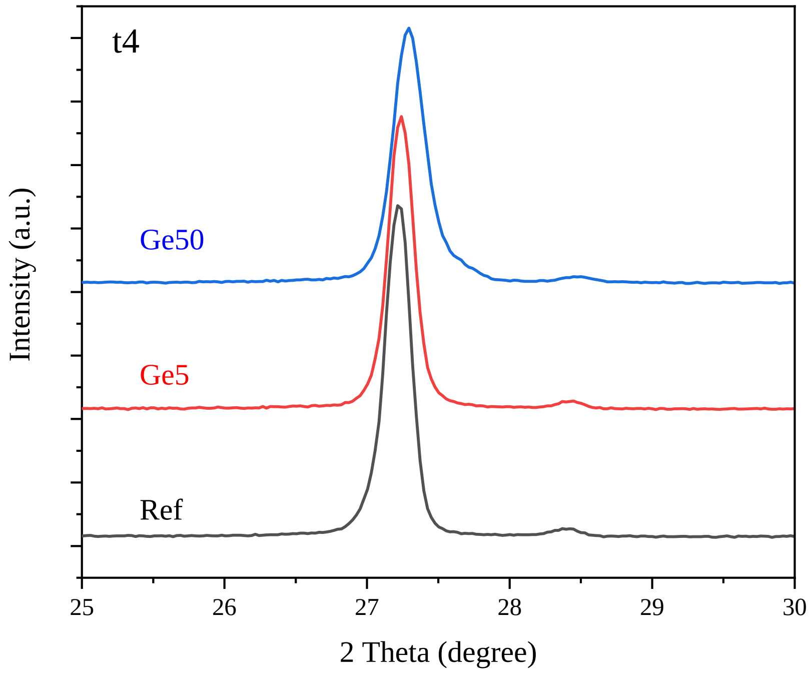

Figure S9. Expanded XRD patterns of (112) peak of samples at t4.

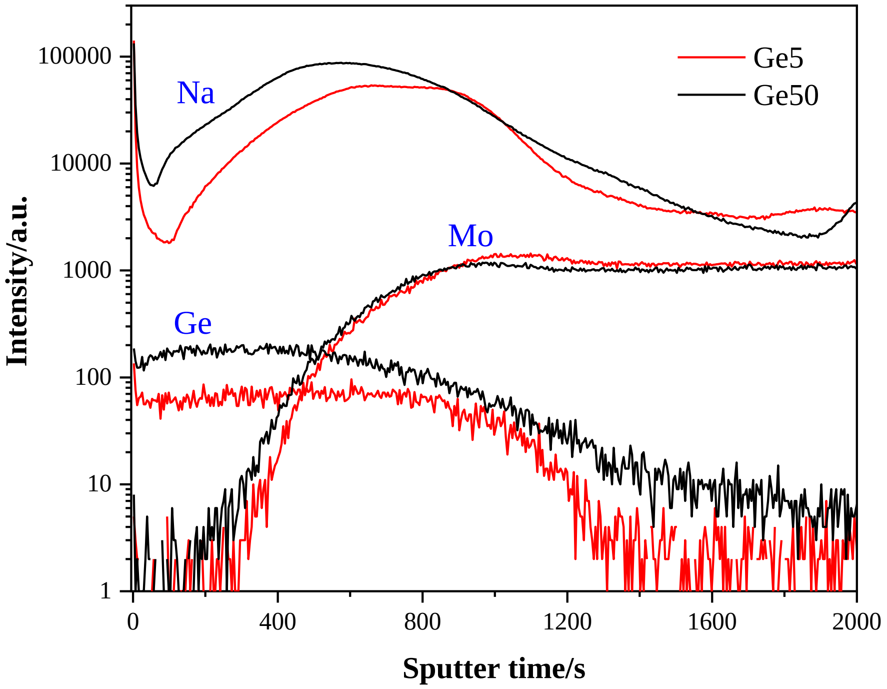

Figure S10. SIMS depth profiles of Ge5 and Ge50.

Table S3. Bulk atomic concentration profile for Ge5 and Ge50 derived from APT.

	**Ge5**	**Ge50**
**Element**	**atomic fraction**	**atomic fraction**
**Cu**	20.821%	20.728%
**Zn**	17.136%	18.595%
**Sn**	14.741%	13.514%
**Se**	42.902%	41.547%
**Ge**	0.097%	0.492%
**O**	4.292%	5.117%
**Na**	0.010%	0.008%

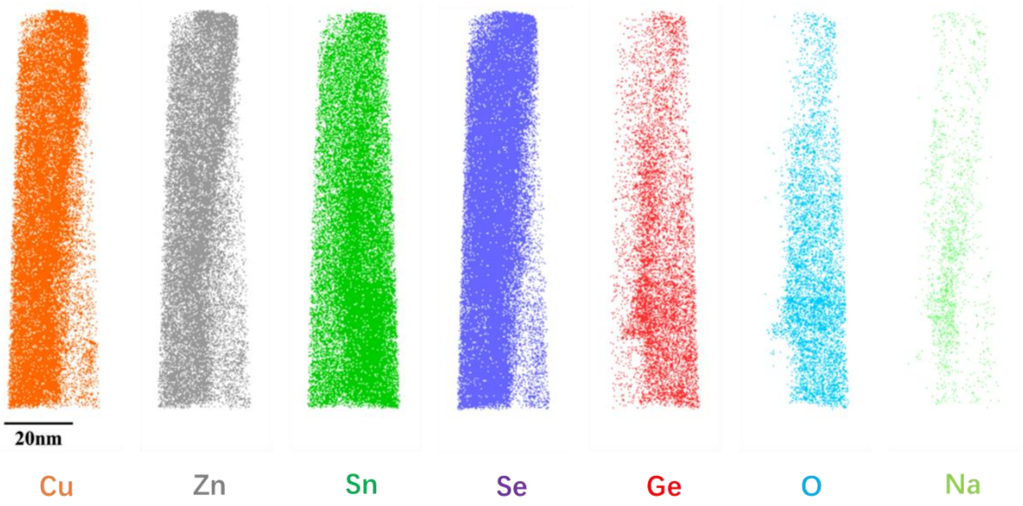

Figure S11. Three-dimension maps of Cu, Zn, Sn, Se, Ge, O and Na from a dataset of Ge5 at t4.
